# Impact of psychiatric disorders on the risk of diabetic ketoacidosis in adults with type 1 diabetes mellitus: a propensity score matching case-control study

**DOI:** 10.1007/s12020-024-04146-y

**Published:** 2025-01-24

**Authors:** Víctor Navas Moreno, Carolina Sager La Ganga, María Sara Tapia Sanchiz, Marta López Ruano, María del Carmen Martínez Otero, Elena Carrillo López, Juan José Raposo López, Selma Amar, Sara González Castañar, Mónica Marazuela, José Alfonso Arranz Martín, Fernando Sebastian-Valles

**Affiliations:** 1 Department of Endocrinology and Nutrition, Hospital Universitario de La Princesa, Instituto de Investigación Sanitaria de La Princesa, Universidad Autónoma de Madrid, Madrid, 28006 Spain; 2 Hospital Universitario de La Princesa, Diego de León 62, Madrid, 28005 Spain

**Keywords:** T1D, Psychiatric disorders, Diabetic ketoacidosis, Mental health

## Abstract

**Purpose:**

This study aims to evaluate the association between psychiatric disorders and diabetic ketoacidosis (DKA) in patients with type 1 diabetes (T1D) treated at a tertiary care hospital.

**Methods:**

A propensity score-matched case-control study was conducted, comprising a total sample of 194 participants (97 DKA cases and 97 controls without DKA). Comprehensive data were collected on clinical, anthropometric, and socioeconomic characteristics, and psychiatric disorders were classified according to international standards.

**Results:**

The mean age of the participants was 47.4 ± 17.7 years, with 55.6% being female. Psychiatric disorders were identified in 16.5% of the study population. The prevalence of psychiatric disorders was significantly higher in DKA cases compared to controls (24.7% vs. 7.2%, *p* < 0.001). Conditional logistic regression models revealed that the association between psychiatric disorders and DKA was not independent of HbA1c levels. Additionally, in HbA1c-stratified analyses, patients with psychiatric disorders developed DKA at lower HbA1c levels compared to controls.

**Conclusion:**

Psychiatric disorders significantly increase the risk of DKA in adults with T1D, particularly among those with less elevated HbA1c levels. These findings highlight the critical importance of addresing psychiatric comorbidities in the management of T1D, given the severe implications and significant healthcare resource utilization associated with DKA.

## Introduction

Type 1 Diabetes Mellitus (T1D) is an autoimmune disease that necessitates stringent management to prevent both acute and chronic complications. Diabetic ketoacidosis (DKA) is a medical emergency characterized by insulin deficiency, leading to hyperglycemia or euglycemia (particularly with the use of newer glucosuric agents), ketonemia, and metabolic acidosis. It is the leading cause of mortality among children and young adults with T1D [1]. In recent years, the incidence of DKA has been increasing, gaining significance due to its associated morbidity, mortality, and the substantial healthcare resources it consumes [2–5].

T1D may coexist with various psychiatric conditions, including affective, anxiety, psychotic, and eating disorders. Additionally, individuals with an initial diagnosis of psychiatric disorders may eventually develop autoimmune diseases such as T1D [6–10]. These psychiatric comorbidities can negatively impact treatment adherence, increasing the risk of DKA due to missed insulin doses or poor glucose management [11]. For example, depression may reduce motivation to adhere to treatment regimens, while anxiety may lead to avoidance behaviors or inappropriate stress management [12]. Eating disorders, such as the deliberate omission of insulin for weight loss (a condition known as diabulimia), significantly elevate the risk of DKA episodes [7, 8].

A multidisciplinary approach that integrates medical treatment with psychological and social support is essential to mitigate the risk of complications in adults with T1D and comorbid psychiatric context. Addressing these conditions from an integrated perspective could not only improve glycemic control but also enhance patient quality of life, thereby reducing the incidence of severe complications such as DKA and the need for hospital admissions [3, 13, 14].

Given these considerations, our objective is to investigate the impact of psychiatric disorders on the risk of hospital admission due to diabetic ketoacidosis in adults with Type 1 Diabetes Mellitus, through a case-control study matched by propensity scores.

## Materials & methods

This retrospective case-control study included all patients diagnosed with DKA who were treated in the Emergency Department of a tertiary hospital in Spain between 2011 and 2024. Eligible cases were required to be aged 15 years or older and meet the following DKA criteria: blood glucose > 200 mg/dL, plasma β-hydroxybutyrate ≥ 3.0 mmol/L and/or urine ketones >2 + , and pH < 7.3 and/or serum bicarbonate < 15.0 mmol/L [1, 15]. Patients who did not meet these criteria or had incomplete personal history information were excluded from the study. The controls consisted of a pool of subjects with T1D who were under follow-up in our outpatient clinics and none of the patients had ever experienced DKA. They were matched with cases by age, sex, disease duration, and presence of diabetic retinopathy, for which a propensity score was calculated, as described in the statistical methods section.

The study adhered to the Strengthening the Reporting of Observational Studies in Epidemiology (STROBE) guidelines and was approved by the [BLINDED] Hospital Research Ethics Committee (Study Number: 5554–06/24), in accordance with the principles of the Declaration of Helsinki.

### Data collection

A comprehensive set of sociodemographic and clinical variables were collected from patients’ medical records, documented during the initial Emergency Department assessment. The collected variables included age, sex, duration of diabetes (in years), body mass index (BMI, kg/m²), presence of diabetic retinopathy, ischemic heart disease, smoking status, insulin dose (IU/kg/day), and chronic treatment with SGLT-2 inhibitors [16].

A psychiatric history was defined as the presence of a formal clinical diagnosis made by a psychiatry specialist within the 6 months preceding the DKA event, accompanied by active pharmacological treatment and regular follow-up. Psychiatric disorders were classified into the following main groups, based on standardized classifications such as the DSM-5: affective disorders, including major depressive disorder and bipolar disorder; anxiety disorders, ranging from generalized anxiety disorder to panic disorder; personality disorders, including borderline and antisocial personality disorders; eating disorders, such as anorexia nervosa and bulimia; and psychotic disorders, including schizophrenia and schizoaffective disorders. The classification considered the comorbidity and the high prevalence of these psychiatric conditions in patients with T1D [17]^(p2)^.

### Socioeconomic status (SES): deprivation index

SES was assessed using the deprivation index, which was calculated based on combined data from the census tract using each participant’s address. This methodology follows an approach similar to that used in previous diabetes research [18–20]. The deprivation index for the entire Spanish territory was based on census section data, combining information on the following variables for each census tract: population employed in manual labor, casual wage-earning population, unemployment, individuals aged 16 and over and those aged 16 to 29 with insufficient education, and primary households without Internet access. In this study, SES will be represented by the deprivation index.

### Statistical analysis

Quantitative variables were represented as mean and standard deviation (SD) or median and interquartile range (IQR), and categorical variables as the number of events and percentage. To address the lack of randomization between study groups and to minimize confounding bias, the 1:1 nearest neighbor propensity score matching method (caliper width 0.015 of the standard deviation [SD] of the logit propensity score [21]) was employed to estimate the effect of treatment on the DKA risk profile. Propensity scores were estimated using logistic regression based on age, sex, duration of diabetes, and presence of retinopathy. Matching was performed without replacement, resulting in the exclusion of 20 patients treated for DKA. The final matched sample comprised 97 individuals in the DKA group, matched 1:1 with control group subjects (*n* = 97) within a 6-month window of the event date. Bivariate differences between these groups were calculated using t-tests or Mann-Whitney U tests for quantitative variables, depending on their distribution, and Chi-square tests for categorical variables.

Additionally, potential confounding factors were assessed based on compliance with confounding criteria in the study variables. The first criterion determined whether the confounder was an independent risk factor for the outcome (DKA), regardless of the intervention. This was evaluated by a significant association between the factor and outcome in the control group. The second criterion assessed whether the confounder was associated with the exposure (psychiatric history), using mean comparison and Spearman correlation analysis [22]. To further investigate the association between the risk of DKA and psychiatric history, a conditional logistic regression model for matched data, adjusted for confounding variables, was performed. A graphical analysis of predicted probabilities was conducted to compare the impact of psychiatric history on HbA1c and DKA risk.

Statistical analysis was performed using STATA 17.0 Basic Edition (Lakeway Drive, TX, USA). Statistical significance was set at *p* < 0.05.

## Results

### Patients

**Fig. 1:**
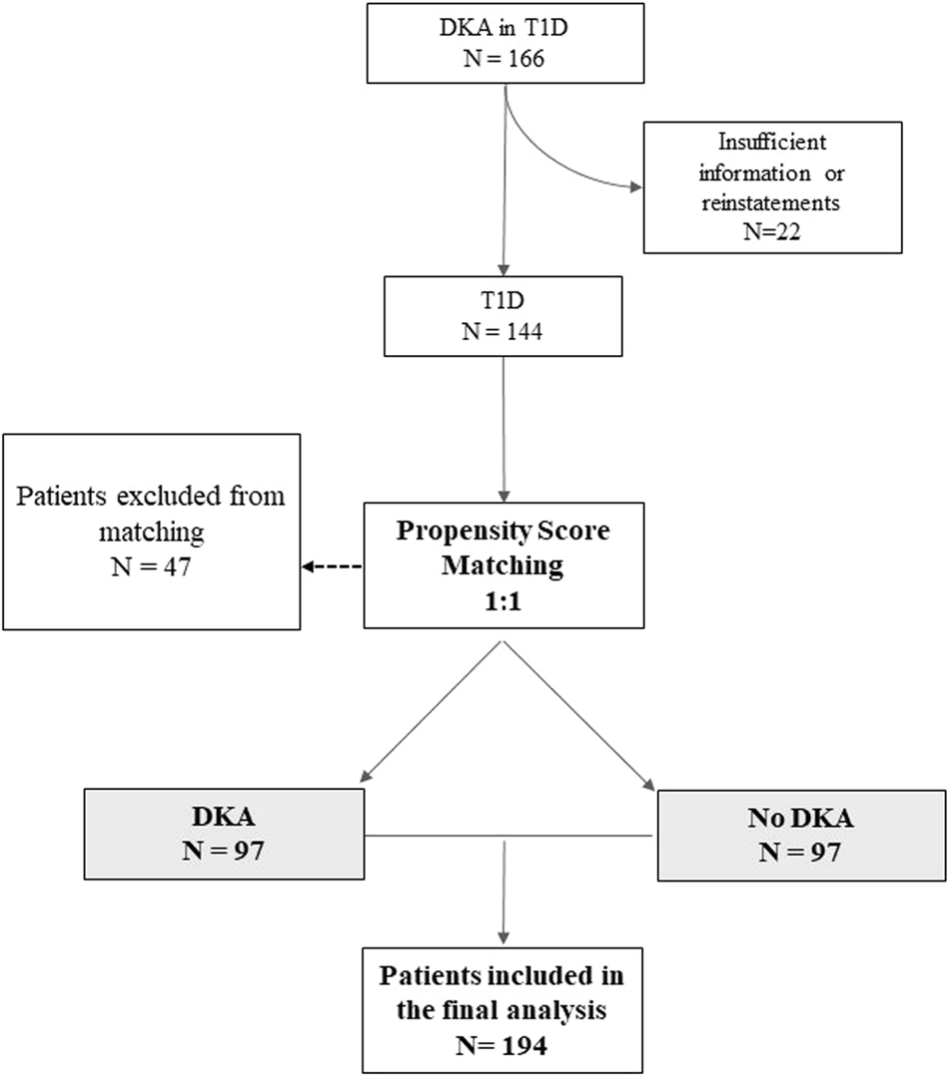
Flowchart diagram. T1D Type 1 Diabetes. DKA Diabetes ketoacidosis

The initial sample consisted of 166 T1D patients who experienced DKA events. After applying the inclusion and exclusion criteria and eliminating readmissions, the sample was reduced to 117 individuals. Following propensity-score matching, 20 cases were excluded, resulting in a final sample of 97 DKA cases and 97 controls, yielding a total of 194 patients with T1D. A flow diagram illustrating this process is presented in Fig. 1.

The mean age of the sample was 47.4 ± 17.7 years, with 109 (55.6%) being women. The mean duration of diabetes was 21.9 ± 12.6 years. The average HbA1c was 8.8 ± 2.3%, with the control group averaging 7.6% compared to 9.9% in the DKA group (*p* < 0.001). Treatment with sodium-glucose cotransporter-2 inhibitors (SGLT2i) was more prevalent among DKA patients (10.3% vs. 2.1% in controls, *p* = 0.017). DKA patients had higher daily insulin doses and a slightly lower BMI (23.4 ± 4.5 vs. 25.0 ± 4.3 kg/m², *p* = 0.011). No significant differences were observed in the deprivation index or smoking habits between cases and controls. The remaining baseline characteristics of the sample are presented in Table 1.

**Table 1:** Sample characteristics

Variable	Obs *n* = 194	NoDKA *n* = 97	DKA *n* = 97	*P*-value
Age (years)	47.4 ± 17.7	47.3 ± 17.4	47.4 ± 18.2	0.978
Sex (woman)	109 (55.6%)	56 (57.7%)	52 (53.6%)	0.563
Duration of diabeyes (years)	21.9 ± 12.6	21.8 ± 11.6	22.2 ± 13.8	0.797
HbA1c (mmol/mol %)	8.8 ± 2.3	7.6 ± 1.5	9.9 ± 2.3	< 0.001
Ischemic heart disease (%)	9 (4.6%)	2 (2.1%)	6 (6.3%)	0.140
BMI (Kg/m²)	24.2 ± 4.4	25.0 ± 4.3	23.4 ± 4.5	0.011
Diabetic retinopathy(%)	35 (17.9%)	17 (17.5%)	18 (18.6%)	0.852
Smoking habit (%)	51 (26.0%)	20 (20.6%)	29 (29.9%)	0.137
Insulin dose IU/kg/day	0.66 ± 0.28	0.58 ± 0.23	0.77 ± 0.30	< 0.001
SGLT-2 inhibitors	12 (6.1%)	2 (2.06%)	10 (10.3%)	0.017
Deprivation Index	−1.18 ± 0.75	−1.21 ± 0.78	−1.15 ± 0.71	0.610

*HbA1c* glycated hemoglobin, *BMI* Body mass index expressed in kilograms/square meter, *SGLT2-inhibitors* Sodium-glucose cotransporter 2 inhibitors

### Psychiatric disorders and risk of diabetic ketoacidosis

**Fig. 2:**
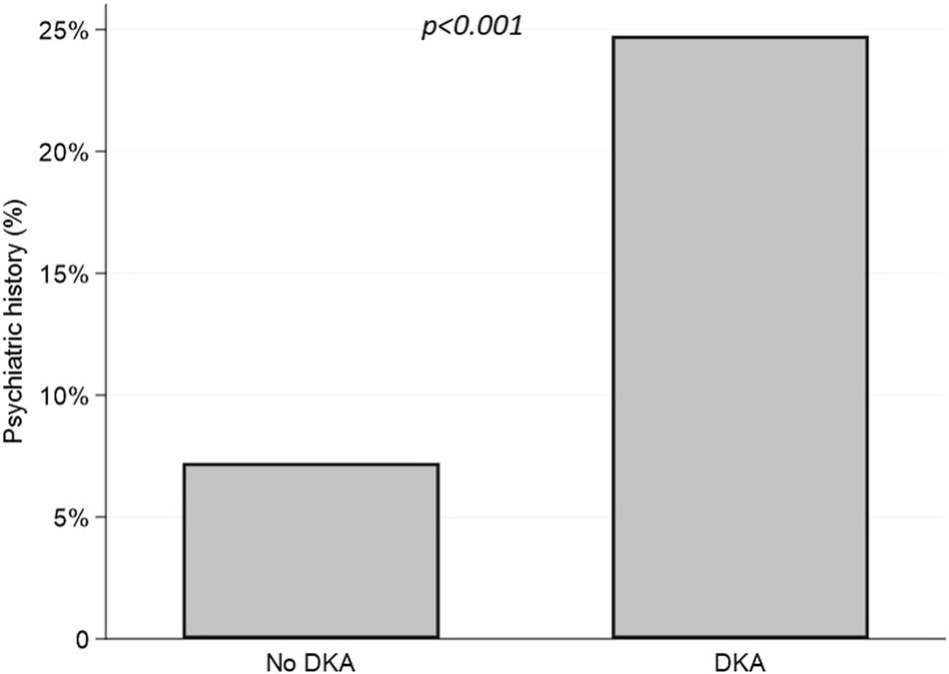
This diagram illustrates the differences in the prevalence of psychiatric history between DKA cases and controls

A higher prevalence of psychiatric disorders was observed in DKA cases compared to controls (24.7% in cases vs. 7.2% in controls, *p* < 0.001) (Fig. 2). Univariate matched analysis revealed statistically significant differences in the prevalence of psychiatric disorders between cases and controls, with an odds ratio (OR) of 3.43 (95% CI: 1.43–9.42, *p* = 0.003). The prevalence of each type of psychiatric disorder within the entire cohort of patients is provided in Supplementary Material S1.

When analysing the specific types of psychiatric disorders in relation to the occurrence of DKA, significant differences were found for anxiety and personality disorders. However, no statistically significant differences were observed for affective disorders or eating disorders (Table 2).

**Table 2:** Types of Psychiatric Disorders Based on the Presence of Diabetic Ketoacidosis

Variable	Obs *n* = 194	No DKA *n* = 97	DKA *n* = 97	*P*-value
Affective disorders (%)	16 (8.2%)	5 (5.1%)	11 (11.3%)	0.117
Anxiety (%)	5 (2.6%)	0 (0%)	5 (5.1%)	0.023
Personality disorder (%)	6 (6.2%)	0 (0%)	6 (6.2%)	0.013
Eating behavior disorder (%)	5 (2.6%)	2 (2.1%)	3 (3.1%)	0.650

*DKA* Diabetic ketoacidosis

Potential confounding factors were considered, including variables associated with DKA that were not part of the matching process, as well as variables related to psychiatric disorders in the control group. The variables associated with DKA are detailed in Table 1. Among the variables analyzed, the only one that showed a significant correlation with psychiatric disorders in the control group was HbA1c, with a correlation coefficient of 0.20. The specific association between HbA1c and psychiatric disorders was investigated using multiple linear regression, which confirmed a statistically significant association (*p* = 0.027), independent of other factors such as insulin dose, BMI, and SGLT-2i treatment. Thus, HbA1c is associated with both the outcome (DKA) and the exposure variable (psychiatric disorders), thereby meeting the first two criteria for confounding. The results of the correlation analysis are presented in Supplementary Material S2.

In the conditional logistic regression model for matched data, it was observed that the effect of psychiatric disorders was neutralized when adjusting for other variables, particularly HbA1c (Supplementary Material S3). However, when a stratified analysis was conducted according to HbA1c levels divided into three tertiles ( < 7.5%, 7.5–9.2%, and > 9.2%), a non-linear relationship between HbA1c and DKA was observed in individuals with psychiatric disorders. Specifically, in the HbA1c tertile of 7.5–9.2%, there was a strong association between psychiatric disorders and DKA (OR 5.06, 95% CI 1.26–20.42, *p* = 0.016), while no significant association was observed in the HbA1c < 7.5% (*p* = 0.513) or > 9.2% (*p* = 0.680) tertiles. To illustrate these results, we have provided in Table 3 the number of patients who experienced DKA based on HbA1c tertiles, along with the presence or absence of psychiatric disorders.

**Table 3:** The relationship between diabetic ketoacidosis and psychiatric disorders based on HbA1c tertiles

HbA1c tertiles	Psychiatric disorders	DKA cases	Odds Ratio	*p*-value
< 7.5% *n* = *63*	No	11 (18.3%)	2.22 CI 95% (0.19–26.8)	*p* = 0.512
	Yes	1 (33.3%)		
7.5–9.2% *n* = *64*	No	21 (42.0%)	5.06 CI 95% (1.26–20.4)	*p* = 0.016
	Yes	11 (78.6%)		
> 9.2% *n* = *61*	No	38 (80.9%)	1.42 CI 95% (0.27–7.50)	*p* = 0.680
	Yes	12 (85.7%)		

The table illustrates the incidence of DKA across different strata of HbA1c, analyzed in relation to the presence of psychiatric disorders. A very low incidence of DKA is observed in the stratum with HbA1c < 7.5%, whereas the proportion rises to 80-85% in the stratum with HbA1c > 9.2%, with no significant differences between patients with psychiatric disorders and controls. However, in the group with suboptimal glycemic control (HbA1c 7.5–9.2%), a higher proportion of DKA is noted among individuals with psychiatric disorders compared to controls (78.6% vs. 42.0%; Odds Ratio = 5.06; *p* = 0.016)

*HbA1c* glycated hemoglobin, *DKA* Diabetic Ketoacidosis

**Fig. 3:**
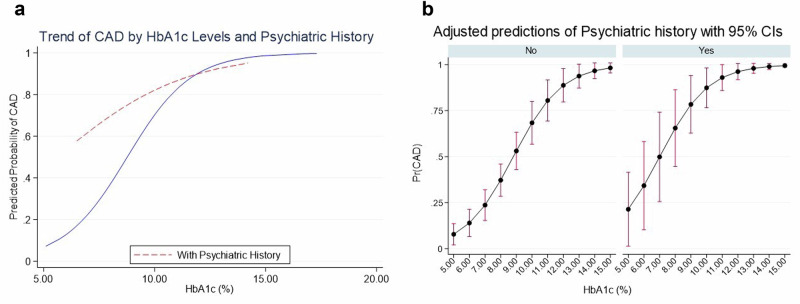
**a**, **b** Graphs illustrating the probability of developing DKA based on HbA1c levels and psychiatric disorders. DKA: Diabetic Ketoacidosis. HbA1c: Glycated Hemoglobin. Figure 3A: Red line (psychiatric history patients): Represents the predicted probability of DKA in subjects with a psychiatric history. Blue line (no psychiatric history patients): Represents the predicted probability of DKA in subjects without a psychiatric history. X-axis (HbA1c%): Represents HbA1c levels. Y-axis (predicted probability of DKA): Shows the predicted probability of DKA, ranging from 0 (0%) to 1 (100%). This graph demonstrates a higher probability of DKA at lower HbA1c levels (5-10%) in the group with a psychiatric history compared to the control group, with this effect diminishing at higher HbA1c levels (> 10%). **b** X-axis (HbA1c%): Represents HbA1c levels. Y-axis (predicted probability of DKA): Shows the predicted probability of DKA, ranging from 0 (0%) to 1 (100%). The slope of the curve is steeper in individuals with a psychiatric history, reaching a 50% probability of DKA at an HbA1c level of 7%, compared to those without a psychiatric history, where the same probability is reached at an HbA1c level of 9%

To further elucidate the modifying effect of psychiatric disorders on the relationship between HbA1c and DKA, a graphical analysis of DKA risk prediction according to HbA1c levels, stratified by psychiatric disorders, was performed (Fig. 3a, b). The probability of DKA increases consistently with HbA1c levels in both groups, but the pattern differed: the highest probability of DKA was observed in the group with psychiatric disorders at lower HbA1c levels.

## Discussion

The relationship between psychiatric comorbidities and T1D is well documented, with evidence indicating an increased risk of complications. To date, this study is the first to examine the impact of psychiatric disorders on the risk of DKA in adult T1D patients, stratified by HbA1c levels. In our cohort, we observed a clear and strong association between DKA and psychiatric disorders.

T1D significantly increases the risk of developing psychiatric disorders throughout childhood, adolescence, and adulthood, as demonstrated by Chen et al. in a large cohort study. The association with affective disorders, anxiety, attention-deficit/hyperactivity disorder in childhood, autism spectrum disorders, and eating disorders is well established [6–9, 12, 23–26], though evidence regarding psychotic disorders remains conflicting [6, 27–29].

Various clusters of psychiatric disorders have been associated with an increased risk of DKA. In a study by Hessler et al., anxiety symptoms (specifically diabetes distress) were linked to worsening glycemic control. However, this was not observed for major depressive disorder [30], aligning with our findings where affective disorders were more common among DKA cases compared to controls, but without reaching statistical significance, unlike anxiety disorders. However, the evidence is mixed, and other studies have shown that depressive symptoms are associated with poorer self-care in diabetes patients compared to diabetes distress symptoms [31]. It is important to distinguish between general anxiety disorders, which were identified in our study, and diabetes distress specifically, as these are distinct entities, with diabetes distress referring to worry or emotional symptoms related to the disease or its management [31, 32].

Low adherence to treatment is the primary precipitating factor for diabetic ketoacidosis [33]. A significant finding in our study is that DKA tends to occur at lower HbA1c levels in patients with psychiatric disorders compared to those without such conditions, with the greatest differences observed in the HbA1c range between 7.5% and 9.2%. It is plausible that patients with HbA1c below 7.5% maintain adequate glycemic control, making the occurrence of DKA unlikely due to proper adherence to treatment. Conversely, those with HbA1c above 9.2% typically exhibit chronically poor adherence to treatment [34], which could explain the lack of significant differences in the incidence of DKA between patients with psychiatric disorders and controls in this HbA1c range.

However, in those with HbA1c levels indicating poor but not extreme glycemic control (7.5–9.2%), we observed a higher prevalence of DKA among those with psychiatric disorders. This could be due to greater treatment manipulation in this group, who may be more prone to skipping doses, fearing hypoglycemia, or making inappropriate adjustments to their treatment. These disruptions in treatment management, particularly in the context of poor chronic control, may increase the risk of precipitating DKA episodes [11, 35, 36]. Nonetheless, these findings need to be confirmed in future studies that evaluate the triggers of DKA in individuals with psychiatric disorders.

In our study, HbA1c was associated with both the exposure factor (psychiatric disorders) and the outcome (DKA), which also produced a change in the observed effect. That is, the higher frequency of DKA in patients with psychiatric disorders cannot be attributed solely to the deterioration of glycemic control, as the risk of DKA in these patients remains elevated even at lower HbA1c levels compared to controls. This suggests that psychiatric disorders act as an effect modifier, increasing the risk of DKA at lower HbA1c levels. These findings emphasize the importance of closely monitoring mental health in T1D patients with psychiatric comorbidities, and ensuring rigorous follow-up to prevent missed doses and optimize glycemic control to avoid DKA episodes.

This study has several limitations. As a case-control design, its ability to establish causal relationships is limited, and the findings should be interpreted as hypothesis-generating rather than definitive conclusions. While the results are consistent and robust, the sample size was small and the study was conducted in a single center, which limits the external validity of the findings. Additionally, data on infections, surgical interventions, trauma, and patient education [3, 5] —classic risk parameters associated with diabetic ketoacidosis—could not be collected, which would have provided a deeper insight into the mechanisms underlying the observed effects. Finally, the criteria used to classify psychiatric disorders excluded those with mild conditions treated in primary care or by psychologists and psychoanalysts, potentially reducing the study’s power.

## Conclusion

Psychiatric disorders were associated with an increased risk of DKA in individuals with T1D. This relationship is not independent of HbA1c levels; rather, psychiatric disorders act as an effect modifier, increasing the risk of DKA at lower HbA1c levels. These findings suggest the need to develop specific therapeutic pathways to prevent DKA in this patient group, considering their clinical particularities.

## Supplementary information

Supplementary material

## Data Availability

No datasets were generated or analysed during the current study.
