# Supplementary material for: Impact of psychiatric disorders on the risk of diabetic ketoacidosis in adults with type 1 diabetes mellitus: a propensity score matching case-control study
Source: Endocrine. 2025 Jan 24;88(1):127–34. doi: 10.1007/s12020-024-04146-y (PMC11933209; doi:10.1007/s12020-024-04146-y)
Supplement: Supplementary file 1 — Supplementary material [file 12020_2024_4146_MOESM1_ESM.pdf]

SUPPLEMENTARY MATERIAL

Supplementary material S1.

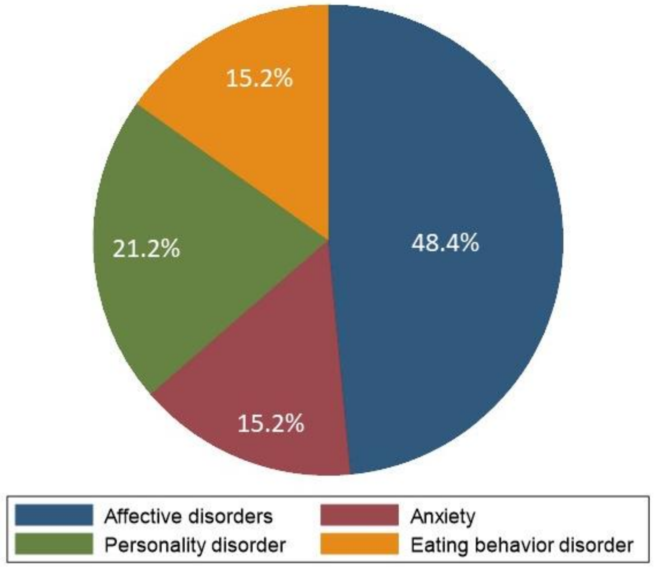

Prevalence of each type of psychiatric disorder within the entire cohort of patients.

Supplementary material S2.

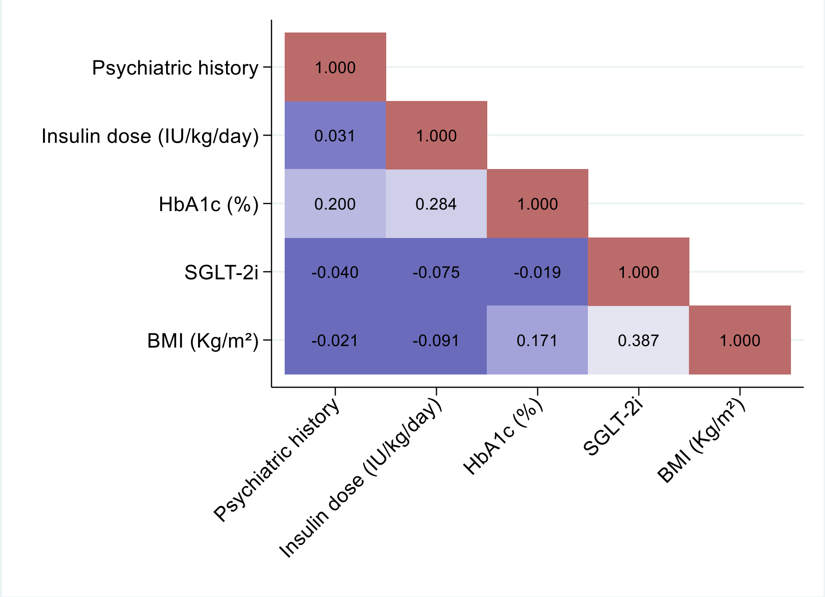

Correlation analysis. The only variable with a significant correlation coefficient of |0.20| is glycated hemoglobin (HbA1c). No other covariates are associated with psychiatric adverse events (AP).

Supplementary material S3.

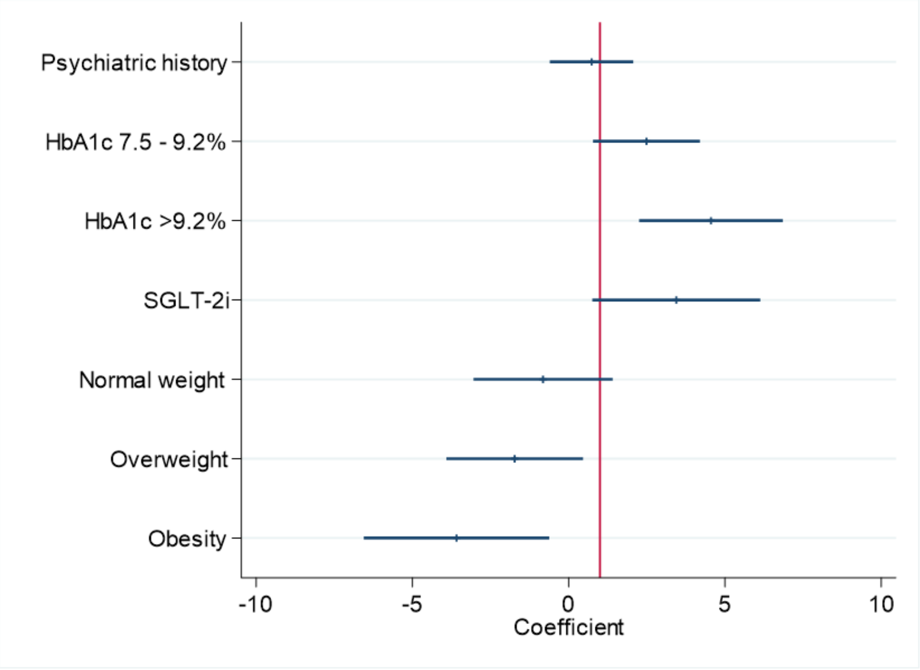

Conditional logistic regression for matched data with HbA1c divided into tertiles, using <7.5% as the reference category, and comparing the two categories of 7.5-9.2% and >9.2%.

A non-linear relationship between HbA1c and DKA is observed in individuals with psychiatric disorders. Specifically, in the HbA1c tertile of 7.5-9.2%, there was a strong association between psychiatric disorders and DKA (OR 5.06, 95% CI 1.26-20.42, p = 0.016), while no significant association was observed in the HbA1c <7.5% (p = 0.513) or >9.2% (p = 0.680) tertiles.
